# Using docking and alchemical free energy approach to determine the binding mechanism of eEF2K inhibitors and prioritizing the compound synthesis

**DOI:** 10.3389/fmolb.2015.00009

**Published:** 2015-03-19

**Authors:** Qiantao Wang, Ramakrishna Edupuganti, Clint D. J. Tavares, Kevin N. Dalby, Pengyu Ren

**Affiliations:** ^1^Division of Medicinal Chemistry, College of Pharmacy, The University of Texas at AustinAustin, TX, USA; ^2^Department of Biomedical Engineering, Cockrell School of Engineering, The University of Texas at AustinAustin, TX, USA; ^3^Graduate Program in Cell and Molecular Biology, The University of Texas at AustinAustin, TX, USA

**Keywords:** free energy calculation, eEF2K inhibitor, docking, molecular dynamics, kinase, drug discovery

## Abstract

A-484954 is a known eEF2K inhibitor with submicromolar IC_50_ potency. However, the binding mechanism and the crystal structure of the kinase remains unknown. Here, we employ a homology eEF2K model, docking and alchemical free energy simulations to probe the binding mechanism of eEF2K, and in turn, guide the optimization of potential lead compounds. The inhibitor was docked into the ATP-binding site of a homology model first. Three different binding poses, hypothesis **1, 2**, and **3**, were obtained and subsequently applied to molecular dynamics (MD) based alchemical free energy simulations. The calculated relative binding free energy of the analogs of A-484954 using the binding pose of hypothesis **1** show a good correlation with the experimental IC_50_ values, yielding an *r*^2^ coefficient of 0.96 after removing an outlier (compound **5**). Calculations using another two poses show little correlation with experimental data, (*r*^2^ of less than 0.5 with or without removing any outliers). Based on hypothesis **1**, the calculated relative free energy suggests that bigger cyclic groups, at R1 e.g., cyclobutyl and cyclopentyl promote more favorable binding than smaller groups, such as cyclopropyl and hydrogen. Moreover, this study also demonstrates the ability of the alchemical free energy approach in combination with docking and homology modeling to prioritize compound synthesis. This can be an effective means of facilitating structure-based drug design when crystal structures are not available.

## Introduction

Computer-based virtual screening (VS) approaches, including docking, pharmacophore, and similarity searching have been proposed and applied at the hit-identification stage of the costly drug discovery process (Singh et al., 2003; Ravindranathan et al., 2010; Kaoud et al., 2012; Wang et al., 2012; Hu et al., 2013; Rea et al., 2013; Sahner et al., 2013; Teli and Rajanikant, 2013; De Luca et al., 2014; Wang et al., 2014; Zhang et al., 2014). These approaches are only effective statistically, e.g., they only provide a meaningful prediction of active compounds in a certain percentile of a large number of samples, rather than information on specific compounds. Such inability hinders their applications in drug discovery processes where relative binding affinities of certain compounds are of interest, for example, in lead-optimization, when tens to hundreds of analogs need to be examined and prioritized for synthesis. Therefore, computational approaches that can correctly predict relative binding affinities are of crucial importance.

Relative free energy of binding as a computable property is a good measure of the binding affinity between two molecules. There is a history of applying physics-based force fields (Jorgensen et al., 1983; Kaminski et al., 2001; Ponder and Case, 2003; Wang et al., 2004; Jiao et al., 2008; Vanommeslaeghe et al., 2010; Ren et al., 2011; Shi et al., 2012; Zhang et al., 2012) or combined quantum mechanics/molecular mechanics (QM/MM) (Raha and Merz, 2005; Wang and Bryce, 2009; Hayik et al., 2010; Min et al., 2010; Wang and Bryce, 2011) methods to predict protein-ligand binding. Specifically, the accuracy of the alchemical free energy approaches in combination with well-tuned force fields has been demonstrated to have a reasonable agreement with experiment results. For example, the typical root mean square differences (RMSD) in hydration free energy of small organic molecules between experiment and prediction are around 1.0 kcal/mol using the commonly used fixed charge force fields, e.g., AMBER/GAFF, CHARMM, and OPLS-AA (Shirts et al., 2003; Shirts and Pande, 2005b; Mobley et al., 2009). While a RMSD of 0.7 kcal/mol has been reported on a smaller scale study of small organic molecules using the multipole-based polarizable AMOEBA force field (Ren et al., 2011). Compared to calculations of hydration free energy, absolute protein-ligand binding free energy calculation is more challenging as the introduction of the protein adds significantly more degrees of freedom to the system (Deng and Roux, 2006; Jayachandran et al., 2006; Mobley et al., 2007; Boyce et al., 2009). Fortunately, the absolute binding free energy is not required to predict the relative binding affinity of two compounds. Instead, the change in binding free energy (ΔΔ*G*) between two compounds is often needed. By constructing an arbitrary thermodynamic path between the two end states (or ligands), the free energy change between them can be obtained by integrating the energy derivative along the path in the so-call thermodynamic integration method (TI) (Kollman, 1993; Shirts and Pande, 2005a). In addition to the TI method which is used in this study, the free energy perturbation (FEP) approach or Bennett acceptance ratio (BAR) are also commonly used in alchemical free energy calculations (Bennett, 1976; Kollman, 1993). In some earlier retrospective studies, the error of the calculated binding free energy is shown to be 1–2 kcal/mol (Jayachandran et al., 2006; Jiao et al., 2008; Michel and Essex, 2008; Boyce et al., 2009; Jiao et al., 2009; Rocklin et al., 2013). Prospective studies, e.g., calculation-driven inhibitor design, have also occasionally been reported in the literature (Jorgensen et al., 2006; Kim et al., 2006; Boyce et al., 2009; Bollini et al., 2011). Building on these recent advances in the theoretical development of the alchemical free energy calculation, we seek here to incorporate this approach to discovery potential inhibitors of eukaryotic elongation factor 2 kinase (eEF2K).

Eukaryotic elongation factor 2 kinase is believed to be a regulator of protein synthesis by phosphorylating the eukaryotic elongation factor 2 (eEF2) protein, which promotes the translocation of the ribosome along mRNA. Upon phosphorylation of eEF2 by the eEF2K, translation elongation is impeded (Carlberg et al., 1990; Kruiswijk et al., 2012). A recent study suggests a regulatory connection between eEF2K and nutrition deprivation. Cells undergoing nutrition deprivation survive by blocking the energy-demanding translation elongation process induced by eEF2K. However, the tumor cells can also exploit this pathway to survive from the metabolic stress (Leprivier et al., 2013). This makes eEF2K a potential target of therapeutic interest in drug discovery. Recently, a selective eEF2K inhibitor A-484954 (we refer to it as compound **3** in this study) with a submicromolar IC_50_ value was reported (Chen et al., 2011). However, the binding mechanism of this compound with eEF2K remains unclear, in part due to the lack of a crystal structure of the kinase.

In this study, we explored the possibility of using *in silico* approaches to design inhibitors for novel targets that do not have crystal structures. Based on a homology model of eEF2K that we built earlier (Devkota et al., 2014), three hypothetical binding poses of A-484954 were first generated from docking. The relative binding free energies of seven novel analogs of A-484954 were calculated for each hypothetical pose using alchemical free energy approach. The predictions were subsequently compared and validated with the experiment IC_50_ values we reported earlier (Edupuganti et al., 2014) although docking and alchemical free energy calculations were performed before the actual chemical and biochemical experiments. The computational results were utilized to prioritize the synthesis of the analog compounds in lead-optimization and provide a better understanding of the molecular interaction between eEF2K and the analogs. Based on the correlation between the calculation and experimental data, the most plausible binding mechanism of the compounds was also discussed.

## Method

### Structure preparation and docking

As no X-ray crystal structure for eEF2K is in the public domain, a homology model has been built in our group (Devkota et al., 2014) using the crystal structures of the alpha-kinase domain of myosin heavy chain kinase A (MHCKA, PDB ID: 3LKM) (Ye et al., 2010) and transient receptor potential (TRP) channels (ChaK) (PDB ID: 1IA9) (Yamaguchi et al., 2001). Based on this 3D model structure, compounds were docked into the ATP binding site of eEF2K using the ChemPLP (Korb et al., 2009) and Goldscore (Jones et al., 1995, 1997) scoring functions in the GOLD5.1 software package.

### Free energy approach

**Figure 1:**
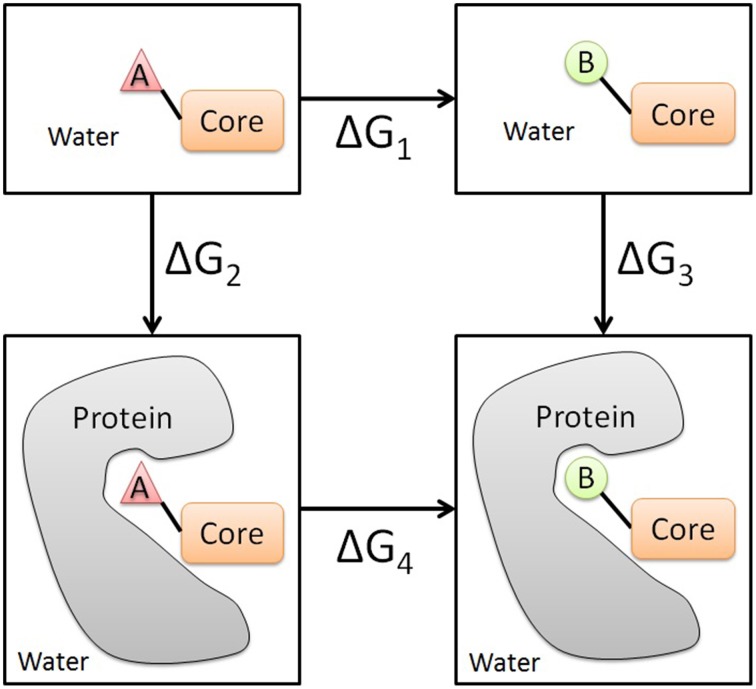
**Illustration of the thermodynamic cycle of the perturbation between the analog compounds A and B**. Core represents the common part between two analogs. The two panels above represent the ligand in the unbound state in water, while the two panels below represent the ligand in the bound state in water.

To evaluate the change in the binding free energy between two analog compounds, a two-step free energy calculation scheme was applied. As shown in Figure 1, the change in the binding free energy between compounds A and B can be calculated either by Δ*G*_3_ − Δ*G*_2_ or by Δ*G*_4_ − Δ*G*_1_. Usually, the later formula is chosen since it is computationally more feasible and practical. To obtain Δ*G*_4_ and Δ*G*_1_, two independent perturbations between compounds A and B need to be conducted in the solvent environment (water in this case) with the protein present and absent respectively. The free energy change ΔG of each perturbation can be evaluated by an alchemical approach, such as the thermodynamic integration (TI) approach used in this study (Kollman, 1993).

For each pair of compounds, an identical protein and octahedral water box were constructed using the *leap* module in the AMBER12 software package (Case et al., 2012). A buffering region of 10 Å is used to solvate the protein-ligand complex and the ligand in the water box. This results a system of ~30,500 atoms for each protein-ligand complexes. The parameters for protein and water are taken from the *ff99SB* force field (Hornak et al., 2006) and the TIP3P water model (Jorgensen et al., 1983) respectively. The ligand parameters are obtained from GAFF (Wang et al., 2004) with the charges fitted from HF/6-31G^*^ calculations. All the simulations were started with a quick minimization to remove the close contacts in the structure, followed by a 50 ps NVT simulation to heat the system up to 300 K and another 50 ps NPT simulation to equilibrate the density of the system, both with a time step of 1 fs. Production NVT simulations of 2–4 ns are then conducted for data collection with a time step of 2 fs. Periodic boundary condition and particle mesh Ewald were used to capture long-range effects. The thermodynamic integration along with a softcore potential implementation (Steinbrecher et al., 2011) in AMBER12 was applied to estimate the free energy. Each perturbation used 11 windows with λ values of 0.01, 0.1, 0.2, 0.3, 0.4, 0.5, 0.6, 0.7, 0.8, 0.9, and 0.99, where electrostatic and van der Waals interactions were perturbed simultaneously. This saves considerable simulation time than perturbing electrostatic and van der Waals interaction separately. All the molecular dynamics (MD) simulations were performed using the AMBER12 software package (Case et al., 2012). Generally, a good convergence in the thermodynamic integration of the ligands in water can be obtained within 1 ns; in contrast, 2–3 ns are normally required for perturbations with the presence of the kinase using the current setting (see data in Supplementary Material). As a result, by using 11 nodes each with two, eight-core Xeon E5-2680 (Sandy Bridge) processors running at 2.7 GHz on Stampede supercomputer in Texas Advanced Computing Center, it takes about 1 day to obtain the free energy change between two ligands.

### Preparation of the compounds

To probe the structure-activity-relationship of the eEF2K inhibitor, a number of analog compounds were designed by replacing different chemical groups at the R1, R2, and R3 sites (Table 1). The analogs were designed based on the predicted importance of the chemical groups in binding from docking and chemical intuition, as well as the ease of chemical synthesis. As a result, three sites of A-484954 (compound **3**) were modified with different substituents. The R1 (cyclopropyl) and R2 (ethyl) moieties were substituted by both smaller and bigger hydrophobic groups, including methyl, ethyl, propyl, cyclopropyl, cyclobutyl, and cyclopentyl. At the R3 site, the CONH_2_ group in compound **3**, which forms a key hydrogen-bond network with the hinge residues of eEF2K, was replaced by a less polar CSNH_2_ group. Details about the synthesis and biochemical experiments can be found in reference (Edupuganti et al., 2014).

**Table 1:** **The analogs tested in free energy calculations**.

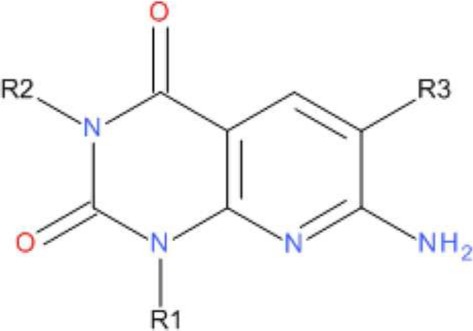			
**Compound**	**R1**	**R2**	**R3**
1	Cyclopropyl	H	CONH_2_
2	Cyclopropyl	Me	CONH_2_
3 (A-484954)	Cyclopropyl	Et	CONH_2_
4	Cyclopropyl	Pr	CONH_2_
5	Cyclopropyl	Et	CSNH_2_
6	Cyclobutyl	Et	CONH_2_
7	Cyclopentyl	Et	CONH_2_
8	Me	Me	CONH_2_

## Results and discussion

### Docking results and the hypothetical binding poses

**Figure 2:**
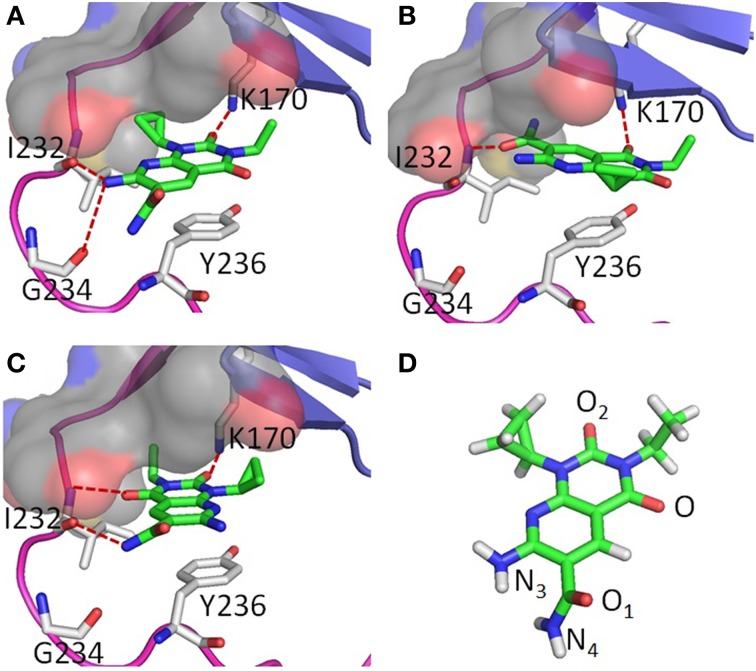
**A-484954 (compound 3) and its hypothetical binding poses to eEF2K homology model structure; (A) is hypothesis 1; (B) is hypothesis 2; (C) is hypothesis 3; and (D) is the structure of A-484954 shown in green sticks**. The hinge region of eEF2K is shown in purple, the glycine-rich loop is shown in blue, and the pocket deep inside the ATP adenine binding site is shown in gray surface.

Two poses were obtained from two independent docking runs (Figures 2A,B). In the first run, the ChemPLP (Korb et al., 2009) function was used as the primary scoring function with the Goldscore (Jones et al., 1995, 1997) function used for rescoring. By ranking with the ChemPLP score, 7 out of the top 10 poses from 20 generic algorithm optimizations agree with hypothesis **1** (Figure 2A), including the top 3 poses. The ChemPLP score for the 7 poses ranges from 68.6 to 67.3, while their corresponding Goldscore ranges from 46.9 to 37.7. In the second run, the two scoring functions were switched, i.e., the Goldscore was used as the primary scoring function, while ChemPLP used to rescore the poses. By ranking the poses with Goldscore, all the top 10 poses out of 20 generic algorithm optimizations agree with hypothesis **2** (Figure 2B) with Goldscores ranging from 53.6 to 52.8, while the ChemPLP scores range from 52.2 to 43.9 for the same poses. It should be noted that the scores between different scoring functions cannot be compared directly. Only the relative scores of the same scoring function can be compared, ideally the higher the better. The results indicate a clear difference in preference between the two scoring functions, i.e., ChemPLP favors hypothesis **1**, while Goldscore favors hypothesis **2**.

In both hypothesis **1** and **2**, three residues, K170, I232, and Y236, play a key role in the binding. Y236 is predicted to hold the ligand via pi-pi stacking with the pyridopyrimidine ring of compound **3** in both binding poses. K170 and I232 all form hydrogen bonds with the ligand, however, differently in the two poses. In hypothesis **1**, the carbonyl group oxygen (O_2_) and amine nitrogen (N_3_) of the ligand form hydrogen bonds with the side chain of K170 and the backbone oxygen atom of I232 respectively. The cyclopropyl moiety of compound **3** fits into the deep cavity of the ATP-binding site of the kinase (shown in gray surface in Figure 2), while the ethyl group goes underneath the glycine-rich loop. In contrast, the hydrogen-bonding network changes to O of compound **3** with K170 and O_1_ with the backbone nitrogen of I232 in hypothesis **2**. The ethyl group still goes underneath the glycine-rich loop, however, the cyclopropyl group is pointing outwards of the pocket. In addition, residue G234 also contributes to the hydrogen bond network with the ligand in hypothesis **1**.

Based on the visual inspection of hypotheses **1** and **2**, we then deduced hypothesis **3** (Figure 2C), which was obtained by manually flipping the ligand around O_2_ in hypothesis **1**. This promotes the O and N_4_ of the ligand forming the hydrogen bond network with the backbone nitrogen and oxygen of I232 respectively. Another difference between hypothesis **3** and **1** is that the position of the ethyl and cyclopropyl group is switched. The ethyl group flipped into the cavity inside the binding pocket and the cyclopropyl went to underneath the glycine rich loop.

To examine the stability of these hydrogen bonds in each poses, all three structures were minimized and relaxed by means of MD simulations for 10 ns. The measurement of the hydrogen bond distance suggests the hydrogen bonds predicted in docking are reasonably stable in general (Table 2). The average distances between the heavier atoms of the hydrogen bonds are generally around 3 Å. However, in hypothesis **3**, the manually docked pose, the hydrogen bond between O_2_ of the ligand and K170 diminished quickly during the MD simulation, resulting an average distance of 6.5 Å (Table 2).

**Table 2:** **Average hydrogen bond distances (in Å) between the kinase and compound 3 from 10 ns molecular dynamics simulations (measured between non-hydrogen atoms)**.

	**Ligand**	**K170**	**I232 (O)**	**I232 (N)**	**G234**
Hypothesis **1**	Compound **3**	2.8	3.2	N/A	2.9
	Compound **5**	2.9	3.4	N/A	5.2
Hypothesis **2**	Compound **3**	3.1	N/A	3.1	N/A
Hypothesis **3**	Compound **3**	6.5	3.3	3.0	N/A

*Data from first 2 ns of each simulation were excluded*.

### Determine the binding mode

To determine the binding pose of compound **3**, the binding free energy change from compound **3** to its analogs are calculated and compared with the experiment IC_50_ values (Table 3). As we have three hypothetical poses, the calculation was repeated for each of them. As free energy methods have been shown to have good accuracy in predicting the relative binding free energy, the hypothetical binding pose which is closer to the true binding mode should give the best correlation between the predicted binding free energy and the experimentally measured binding affinity.

**Table 3:** **Calculated binding free energy change (ΔΔ*G*, kcal/mol) of the analogs against A-484954 and their experimental IC_50_ values (μM)**.

**Compound**	IC^*^_50_	**ΔΔ*G* w.r.t. compound 3**		
		**Hypothesis 1**	**Hypothesis 2**	**Hypothesis 3**
1	6.6	2.0 ± 0.3	1.6 ± 0.5	1.1 ± 0.5
2	6.1	1.5 ± 0.2	0.5 ± 0.1	−0.5 ± 0.1
3 (A-484954)	0.42	0	0	0
4	0.93	0.7 ± 0.3	2.9 ± 0.3	0.3 ± 0.4
5	>25	0.4 ± 0.3	6.9 ± 0.6	5.8 ± 0.4
6	N/A	−1.1 ± 0.4	N/A	N/A
7	N/A	−2.6 ± 0.4	N/A	N/A
8	>25	3.1 ± 0.4	−0.6 ± 0.3	0.7 ± 0.5

A positive change in ΔΔG indicates a cost in energy of the mutation from compound ***3***, which suggests a decrease in potency.

* *Details about the measurement can be found in ref (Edupuganti et al., 2014)*.

It should be noted that the binding free energy calculated here is the relative binding free energy, i.e., the cost in free energy by substituting compound **3** with another compound. A positive value suggests a cost in energy, thus the substituted compound (i.e., **3**) should be a stronger binder than the other. Conversely, a negative value means the substituent binds better. For example, in Table 3, from compounds **3** to **1**, the predicted change in binding free energy is 2.0 kcal/mol based on hypothesis **1**, thus compound **3** is predicted to have a higher binding affinity than compound **1**.

To determine the binding mode of compound **3**, each of the hypothetical binding poses need to be justified. In hypothesis **2** (Figure 2B), for example, the CONH_2_ group forms a hydrogen bond with the hinge residue of eEF2K, which implies that the oxygen atom may be important in binding. Substituting CONH_2_ (compound **3**) with CSNH_2_ (compound **5**) yield a 6.9 kcal/mol energy cost based on the free energy calculation (Table 3). This is in accordance with the trend of measured IC50 values, which increases from 0.42 μM to >25 μM for compound **5**. However, when the cyclopropyl group in compound **3** is substituted by methyl (compound **8**), an increase in affinity would be expected as the cyclopropyl is exposure to solvent in hypothesis **2**, substituting it with a smaller hydrophobic group might be more favorable. Not surprisingly, the calculated free energy change based on this pose suggests this is a favorable substitution, given a free energy change of -0.6 kcal/mol. However, the measured IC_50_ value indicates this is an unfavorable change, where the IC_50_ of compound **8** is at least 50-fold weaker than compound **3**. Nonetheless, such results demonstrate a good prediction of the free energy change, but possibly indicate a bad binding pose predicted in hypothesis **2**.

Similarly, when the ethyl group in compound **3** was substituted by the methyl group (compound **2**), the free energy calculated based on hypothesis **3** suggested it is a favorable change (-0.5 kcal/mol), while the calculation based on hypothesis **1** suggested an unfavorable change (1.5 kcal/mol). Comparing the IC_50_ value of 6.1 μM of compound **2** with 0.42 μM of compound **3**, it is clear that the prediction based on hypothesis **1** is more accurate. To make a conclusion, a systematic comparison of the correlation between the calculated relative free energy and the experimental IC_50_ values are conducted.

To facilitate the comparison, relative ln(IC_50_) values were calculated based on the original experiment IC_50_ data. For each compound, the natural logarithms were first obtained. Then the relative values were calculated against compound **3**, to be consistent with the free energy calculations. As the IC_50_ values for compounds **5** and **8** have been measured only to be >25 μM, they were all considered to have an IC_50_ value of 25 μM to simplify the comparison. For compounds **6** and **7**, due to the difficulties in synthesis, they were not included in the comparison. This results six data points for each of the hypothetical binding poses.

**Figure 3:**
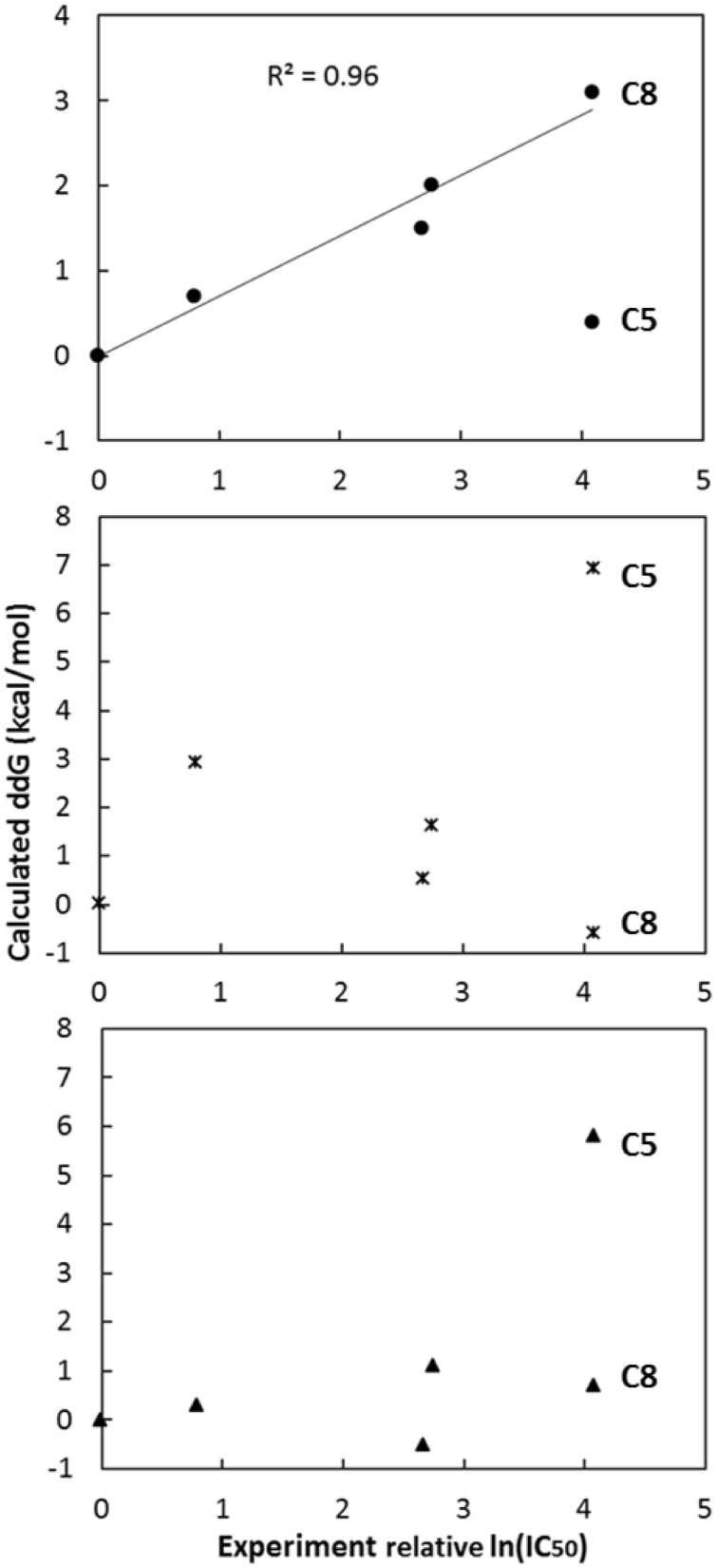
**Plots of the calculated relative binding free energy and the experimental relative ln(IC_50_) values, i.e., compounds X—compounds 3 (*X* = 1, 2, 4, 5, 8), use each of the three hypothetical binding poses (dots for hypothesis 1, asterisks for hypothesis 2 and triangles for hypothesis 3)**. After removing one outlier (either compound **5** or **8**), the best *r*^2^ correlation coefficient for hypothesis **1**, **2**, and **3** are 0.96 (removing compound **5**), 0.42 (removing compound **8**), and 0.49 (removing compound **8**; removing compound **5** yields a *r*^2^ of 0.13), respectively, while the Kendall's τ rank correlation are 0.97, 0.69 and 0.55 (0.41), respectively, by removing the same compound in *r*^2^ calculation.

As shown in Figure 3, the calculated relative binding free energy was plotted against the experimental IC_50_ values. A good correlation between the calculation and experiment is observed for hypothesis **1** in general (Figure 3). In contrast, neither hypothesis **2** nor **3** shows strong correlation between the predictions and the actual experiment measurements. If all six data points were considered, the *r*^2^ correlation coefficient for hypothesis **1**, **2**, and **3** are 0.37, 0.08, and 0.31 respectively, while the Kendall's τ rank correlation coefficient are of 0.55, 0.14, and 0.55. All are not meaningful. However, if one outlier (compound **5**) is removed in hypothesis **1**, the best *r*^2^ coefficient becomes 0.96. In contrast, the best *r*^2^ coefficient of hypothesis **2** and **3** after removing any outliers are 0.42 (removing compound **8**) and 0.49 (removing compound **8**; removing compound **5** yields a *r*^2^ of 0.13) respectively. The Kendall's τ rank correlation coefficient becomes 0.97, 0.69, and 0.55 (0.41) by removing the same compounds. With such a good correlation for hypothesis **1**, it is likely that this pose is closer to the true binding mode of compound **3** in eEF2K.

### Ranking and prioritizing the compounds

It is shown that hypothesis **1** may be the closest to the true binding mode of compound **3** with eEF2K in previous section. The relative potency of five (out of six) compounds is correctly predicted. The correlation between the prediction and experimental measurement of the five compounds is rather good, with an *r*^2^ correlation coefficient of 0.96 and a Kendall's τ rank correlation of 0.97 (Figure 3). The calculation correctly predicted the structure-activity-relationship of the R1 and R2 sites of compound **3** (Tables 1, 3). At the R1 site, the calculation suggested that bigger hydrophobic groups, for example cyclobutyl and cyclopentyl tested in calculation, are generally more potent than smaller groups in the order of cyclopentyl (compound **7**) > cyclobutyl (compound **6**) > cyclopropyl (compound **3**) > methyl (compound **8**). The change in binding free energy from compound **3** to compounds **7**, **6**, and **8** are −2.6, −1.1, and 3.1 kcal/mol respectively. A negative change in the binding free energy suggests a favorable change, i.e., a more potent compound, while a positive change suggests an unfavorable change, i.e., a less potent compound. The prediction for the change from compound **3** to compound **8** has been verified by experimental IC_50_ values of 0.42 and >25 μM for the two compounds respectively. However, for compound **6** and compound **7**, experimental results are not obtained due to the difficulties in synthesis of the two compounds. Another compound, which uses ethyl to substitute cyclopropyl at the R1 site, has an IC_50_ value between 1 ~ 2 μM (Edupuganti et al., 2014). This is in accordance with our predicted structure-activity-relationship.

At the R2 site, the calculated free energy suggested that the ethyl group of compound **3** is the optimal group. Replacing it with bigger or smaller hydrocarbon groups all lowers the potency of the compound. The calculated binding free energy change from substituting ethyl with hydrogen (compound **1**), methyl (compound **2**) and propyl (compound **4**) are 2.0, 1.5, and 0.7 kcal/mol, respectively. This is in an excellent agreement with the experimental IC_50_ values of 0.42, 6.6, 6.1, and 0.93 μM for compounds **3**, **1**, **2**, and **4**.

**Figure 4:**
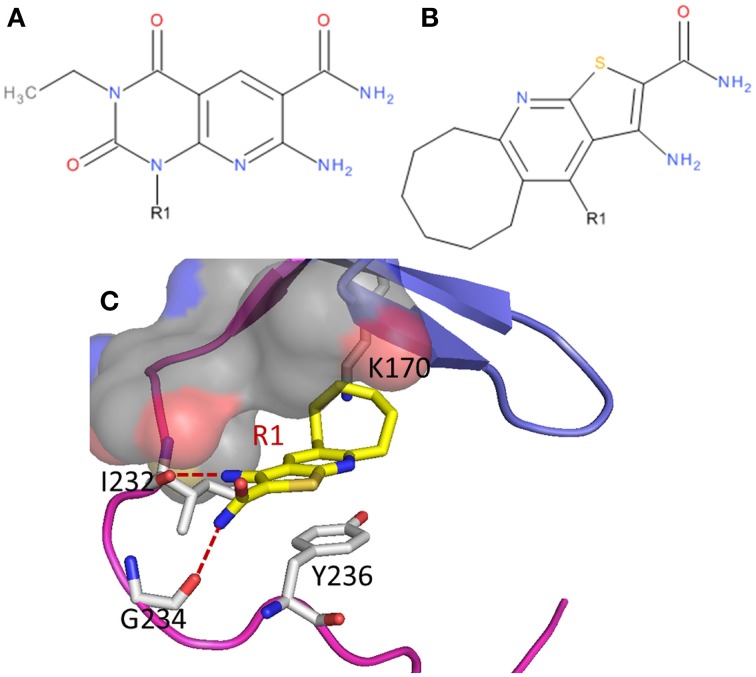
**Two eEF2K inhibitors, (A) A-484954 and (B) compound 2 in the reference (Lockman et al., 2010), (C) in complex with eEF2K with the conformations of hypothesis 1**.

An earlier study (Lockman et al., 2010) also reported a compound (Figure 4B), which has similar scaffold as compound **3**, favors relatively bigger groups (e.g., furan and isobutyl) than smaller groups (e.g., hydrogen atom and methyl) at R1 in general. After docking this compound to eEF2K in reference to the binding poses of compound **3** in hypothesis **1** and **2**, two binding poses are obtained. We then calculated the relative binding free energy upon the substitution of the hydrogen atom with a furan group at R1 of the compound (Figure 4). The calculated changes in binding free energy are of −0.8 ± 0.4 and −2.6 ± 0.3 kcal/mol for the two poses, respectively. Given the experimental IC_50_ values of 2.5 and 0.64 μM of the compounds with hydrogen and furan at R1, respectively, the calculated results based on hypothesis **1** (ΔΔ*G* = −0.8 ± 0.4 kcal/mol) is clearly better correlated with the 4-fold increase in binding affinity measured in experiment.

### Problem in free energy calculation between compounds 3 and 5

As discussed in previous sections, the calculated change in the binding free energy from compound **3** to **5** in hypothesis **1** was the only outlier in comparison with the experimental IC_50_ values (Figure 3). However, the independent MD simulations of compounds **3** and **5** clearly indicates that the later lost one key hydrogen bond between the N_3_ atom of the compound and the Gly234 residue in the hinge region of the kinase (Figure 2 and Table 2). A short hydrogen bond distance of 2.9 Å between Gly234 and compound **3** suggests a strong interaction between the kinase and the ligand. However, after the CONH_2_ group is substituted with CSNH_2_, this hydrogen bond is completely lost, given an average distance of 5.2 Å in the MD simulation of compound **5** (Table 2). Quantum mechanical calculations (HF/6-31G^*^) for compounds **3** and **5** suggest that the substitution of CONH_2_ with CSNH_2_ is associated with significant change on the charge distribution of the atoms nearby. As a result, the restrained electrostatic potential (RESP) method fitted partial charges of the neighbor NH_2_ group, as well as the carbon atoms, are less polar in compound **5** comparing to the charges in compound **3**. The calculated electrostatic interaction energy between compound **5** and eEF2K is about 9.1 kcal/mol less favorable than that of compound **3**, while the contribution from vdWs interaction of compounds **5** and **3** are about the same, of −40.5 and −40.8 kcal/mol, respectively (averaged energy from MD simulations). This shift in energy is in accordance with the observed shift in hydrogen bond distances. These evidences all suggest that compound **5** may be a weaker binder than **3**. The failure of the free energy calculation between compounds **3** and **5** might be a combination of many factors, e.g., the force field and the free energy method. More studies are needed before a conclusion can be drawn. This, however, is out the scope of our study.

## Conclusion

In this study, the binding mechanism of a known eEF2K inhibitor (Chen et al., 2011) (compound **3**) has been studied using docking and alchemical free energy approaches in conjunction with experimental measurements. The inhibitor was firstly docked into the ATP-binding site of a homology model (Devkota et al., 2014) we built earlier. Then, three different binding poses, hypothesis **1**, **2**, and **3**, were used to predict the structure-activity-relationship (SAR) of the inhibitor. The calculated relative binding free energy of analogs of compound **3** using the pose in hypothesis **1** shows a good correlation with the experimental IC_50_ values, giving an *r*^2^ coefficient of 0.96 after removing a suspicious outlier (compound **5**). Calculations using another two poses merely show correlations with experiment, given the *r*^2^ coefficients of <0.5 either or not remove any single outliers (Figure 3). Based on hypothesis **1**, further free energy calculations suggest bigger cyclic groups, e.g., cyclobutyl and cyclopentyl, at R1 might be more favorable in binding than smaller groups, e.g., cyclopropyl and hydrogen. This is in accordance with previous study of an eEF2K inhibitor which has similar core scaffold as compound **3** (Lockman et al., 2010). At R2, the ethyl group might be close to the optimal size, as either longer or shorter side chains do not increase the potency of the compound. Moreover, this study also demonstrates the ability of the alchemical free energy approach in prioritizing the compound synthesis in combination with docking and homology modeling when experimental protein structures are not available, suggesting an alternative way to reduce the dependence of crystal structures in structure-based drug design, as well as the possibility to reduce the total number of compounds that need to be synthesized and tested.

### Conflict of interest statement

The authors declare that the research was conducted in the absence of any commercial or financial relationships that could be construed as a potential conflict of interest.

## Supplementary material

Detailed free energy results is provided in the Supporting Material, including the perturbation from bounded (protein-ligand complex in water) and unbounded (ligand in water) states, as well as the convergence of the simulations.
