# Supplementary material for: Using docking and alchemical free energy approach to determine the binding mechanism of eEF2K inhibitors and prioritizing the compound synthesis
Source: Front Mol Biosci. 2015 Mar 19;2:9. doi: 10.3389/fmolb.2015.00009 (PMC4429643; doi:10.3389/fmolb.2015.00009)
Supplement: Supplementary file 1 [file DataSheet1.DOCX]

Supporting Material

Table S1. The convergence of the calculated free energy change (kcal/mol) in each perturbation of the three Hypothetic binding poses (N.B. This is not the change of the binding free energy). The average value of every 0.5 ns is shown. The final value is estimated based on the average value of the 0.5 ns averages. The first one or two 0.5 ns values were excluded if they were apparently different from the subsequent values. The error was estimated based on the standard deviation of all the 0.5 ns values used in the estimation of the final value.

| Perturbation | 0-0.5 ns | 0.5-1 ns | 1-1.5 ns | 1.5-2 ns | 2-2.5 ns | 2.5-3 ns | 3-3.5 ns | 3.5-4 ns | Est. |
| --- | --- | --- | --- | --- | --- | --- | --- | --- | --- |
| $\Delta G_{1}$ | Unbound state | | | | | | | | |
| 3 to 2 | -29.9 | -29.8 | -29.8 | -29.8 |  |  |  |  | -29.8 ±0.03 |
| 2 to 1 | 11.2 | 11.1 | 11.1 | 11.1 |  |  |  |  | 11.1 ±0.02 |
| 2 to 8 | -102.1 | -102.1 | -102.1 | -102.1 |  |  |  |  | -102.1 ±0.01 |
| 3 to 4 | -10.2 | -10.2 | -10.3 | -10.2 |  |  |  |  | -10.2 ±0.05 |
| 3 to 5 | -188.5 | -188.7 | -187.8 | -187.6 | -187.6 | -187.7 |  |  | -187.7 ±0.07 |
| 3 to 6 | -21.6 | -21.2 | -21.2 | -21.0 |  |  |  |  | -21.1 ±0.08 |
| 3 to 7 | -14.3 | -14.1 | -14.3 | -14.2 |  |  |  |  | -14.2 ±0.08 |
|  |  |  |  |  |  |  |  |  |  |
| $\Delta G_{4}$ | Bound state: Hypothesis **1** | | | | | | | | |
| 3 to 2 | -28.0 | -28.2 | -28.6 | -28.3 |  |  |  |  | -28.3 ±0.2 |
| 2 to 1 | 12.5 | 11.8 | 11.5 | 11.5 |  |  |  |  | 11.6 ±0.1 |
| 2 to 8 | -100.5 | -100.8 | -100.7 | -99.9 |  |  |  |  | -100.5 ±0.2 |
| 3 to 4 | -7.8 | -7.9 | -9.2 | -9.7 | -9.8 | -9.2 |  |  | -9.5 ±0.3 |
| 3 to 5 | -187.4 | -187.1 | -187.7 | -186.9 | -187.0 | -187.5 |  |  | -187.3 ±0.3 |
| 3 to 6 | -22.5 | -21.8 | -22.1 | -22.5 |  |  |  |  | -22.2 ±0.3 |
| 3 to 7 | -16.5 | -17.3 | -16.8 | -16.7 |  |  |  |  | -16.8 ±0.3 |
|  |  |  |  |  |  |  |  |  |  |
| $\Delta G_{4}$ | Bound state: Hypothesis **2** | | | | | | | | |
| 3 to 2 | -30.7 | -29.3 | -29.3 | -29.4 |  |  |  |  | -29.3 ±0.04 |
| 2 to 1 | 12.7 | 11.9 | 11.8 | 12.5 |  |  |  |  | 12.2 ±0.4 |
| 2 to 8 | -103.0 | -103.2 | -103.5 | -103.0 |  |  |  |  | -103.2 ±0.2 |
| 3 to 4 | -5.3 | -4.8 | -5.8 | -6.9 | -7.0 | -7.5 | -7.8 | -7.5 | -7.3 ±0.3 |
| 3 to 5 | -180.7 | -181.1 | -182.0 | -180.8 |  |  |  |  | -181.1 ±0.5 |
|  |  |  |  |  |  |  |  |  |  |
| $\Delta G_{4}$ | Bound state: Hypothesis **3** | | | | | | | | |
| 3 to 2 | -30.5 | -30.2 | -30.4 | -30.2 |  |  |  |  | -30.3 ±0.1 |
| 2 to 1 | 11.9 | 12.7 | 13.2 | 12.8 | 12.7 | 12.8 |  |  | 12.7 ±0.4 |
| 2 to 8 | -100.4 | -101.1 | -101.1 | -100.9 |  |  |  |  | -100.9 ±0.3 |
| 3 to 4 | -7.6 | -8.9 | -9.3 | -9.6 | -10.4 | -10.1 | -10.2 | -9.5 | -9.9 ±0.4 |
| 3 to 5 | -182.1 | -181.9 | -182.6 | -182.3 |  |  |  |  | -182.2 ±0.3 |
